# Time lag effect on malaria transmission dynamics in an Amazonian Colombian municipality and importance for early warning systems

**DOI:** 10.1038/s41598-023-44821-0

**Published:** 2023-10-30

**Authors:** William Gonzalez-Daza, Rafael Jose Vivero-Gómez, Mariano Altamiranda-Saavedra, Renata L. Muylaert, Victor Lemes Landeiro

**Affiliations:** 1 Programa do Pós-Graduação em Ecologia e Conservação da Biodiversidade, Departamento de Biociências, Universidade Federal de Mato Grosso, Cuiabá, MT 78060-900 Brazil; 2 Grupo de Microbiodiversidad y Bioprospección, Laboratorio de Biología Celular y Molecular, Universidad Nacional de Colombia Sede Medellín, Street 59A #63-20, 050003 Medellín, Colombia; 3 Programa de Estudio y Control de Enfermedades Tropicales-PECET, Universidad de Antioquia, Calle 62 No. 52-59 Laboratorio 632, Medellín, Colombia; 4 Grupo de Investigación Bioforense, Tecnológico de Antioquia Institución Universitaria, Medellín, Colombia; 5 Molecular Epidemiology and Public Health Laboratory, School of Veterinary Science, Massey University, Palmerston North, New Zealand; 6 Departamento de Botânica e Ecologia, Instituto de Biociências, Universidade Federal de Mato Grosso, Cuiabá, MT 78060-900 Brazil

**Keywords:** Computational biology and bioinformatics, Ecology, Climate sciences, Diseases

## Abstract

Malaria remains a significant public health problem worldwide, particularly in low-income regions with limited access to healthcare. Despite the use of antimalarial drugs, transmission remains an issue in Colombia, especially among indigenous populations in remote areas. In this study, we used an SIR Ross MacDonald model that considered land use change, temperature, and precipitation to analyze eco epidemiological parameters and the impact of time lags on malaria transmission in La Pedrera—Amazonas municipality. We found changes in land use between 2007 and 2020, with increases in forested areas, urban infrastructure and water edges resulting in a constant increase in mosquito carrying capacity. Temperature and precipitation variables exhibited a fluctuating pattern that corresponded to rainy and dry seasons, respectively and a marked influence of the El Niño climatic phenomenon. Our findings suggest that elevated precipitation and temperature increase malaria infection risk in the following 2 months. The risk is influenced by the secondary vegetation and urban infrastructure near primary forest formation or water body edges. These results may help public health officials and policymakers develop effective malaria control strategies by monitoring precipitation, temperature, and land use variables to flag high-risk areas and critical periods, considering the time lag effect.

## Introduction

Malaria is a parasitic disease that continues affecting millions of people worldwide, especially those living in low-income regions with limited access to healthcare; an estimated 247 million cases of malaria occurred globally in 2021, resulting in 619,000 deaths^1^. In South America, the Amazon region is one of the most affected areas, with countries such as Brazil, Colombia, Peru, and Venezuela reporting the highest number of cases^2^. Malaria is particularly prevalent in the tropical regions of these countries, where the climate and geography provide optimal conditions for the *Anopheles* species that transmit the disease^3^.

According to the National Institute of Health of Colombia (INS), Colombia had a total of 81,363 cases reported for 2020, of these cases, 49.7% were caused by *P. vivax*, and 49.5% were caused by *P. falciparum*^4^. Despite the fact that 91% of Colombia is susceptible to malaria transmission, a big proportion of malaria cases are located in the Pacific region and are mainly caused by *Plasmodium falciparum*^5^. On the other hand, in most municipalities at risk, including the entire Amazon area, *Plasmodium vivax* is the predominant cause of malaria^6^. This area is home to a high diversity of *Anopheles* mosquito species, being the most common *A. darling*, *A. nuneztovari* and *A. albimanus*^7^, surprisingly, with higher species number in deforested areas^8–12^.

Despite efforts to control malaria transmission, including the use of antimalarial drugs and long-lasting insecticidal nets (LLINs), Colombia has not seen a decreasing trend in malaria cases in recent years and remains a major public health problem, especially among indigenous populations living in remote areas with limited access to healthcare^13,14^. In fact, The Annual Parasite Index (IPA) observed for 2020 was 8.4 cases per 1000 inhabitants at risk, one of the highest incidences in recent years after that obtained in 2010 (11.5 cases per 1000 inhabitants at risk) and in 2019 (10.01 cases per 1000 inhabitants at risk)^5^. This is in contrast to other countries in the region, such as Brazil, which have reported a decreasing trend in malaria cases in recent years^15^. The persistence of malaria in Colombia underscores the need for continued efforts to strengthen surveillance and control measures, particularly in high-transmission areas^16^.

In order to understand the conditions that drive malaria transmission in Colombia, it is important to consider that malaria transmission is influenced by a complex array of factors, including climate, land use, and human behavior^3,17,18^. Deforestation and climate change are two key drivers that raise malaria transmission risk, particularly in tropical regions such as the Amazon basin^19–22^. Deforestation creates ideal breeding habitats for *Anopheles* mosquitoes by creating standing water and removing the forest canopy that regulates local temperature and humidity^12,23,24^. Moreover, deforestation can lead to population displacement and migration, which can further increase malaria transmission by introducing the disease to new areas^9^.

Although there have been various studies conducted in Colombia that establish a relationship between climate, land use, and malaria risk^25–28^, there are still significant gaps that need to be addressed^16^. These gaps can be filled by implementing better prevention and control measures in areas previously classified as moderate and high risk and by contributing to the development of early warning systems^29^. Besides, is critical the inclusion of eco-epidemiological information in finer spatial and temporal scales^30^. The coarse temporal scale in the annual models fail to account for the seasonal variations, which play a vital role in malaria outbreaks^31^. Moreover, finer scales than at the municipal level need to be considered since malaria transmission is context-dependent, and the landscape can vary significantly within a single municipality^32^. Lastly, it should be noted that the majority of research in Colombia have primarily focused on the Pacific region due to higher outbreaks numbers^33,34^, however, given its high likelihood for malaria transmission, it is imperative to conduct malaria research in the Amazon biome^2^.

On the other hand, the use of mechanistic models such as SIR Ross Macdonald models serve as a valuable tool for evaluating the impact of malaria control and prevention measures, enabling the analysis of various scenarios and identification of eco-epidemiological parameters that exert the greatest influence on disease transmission^35^. The SIR models are based on the assumption that individuals can be classified into three compartments: susceptible (S), infectious (I), and recovered (R) and how the number of individuals in each compartment changes over time due to malaria transmission and recovery^36^. Some variations of the SIR model can incorporate mosquitoes compartments as well and environmental variables such as temperature and rainfall, which affect the mosquito population and the malaria transmission rate^37^. Nevertheless, its use in exploring the relationship between land use and malaria is relatively scarce. Besides, incorporating these variables into mathematical models of malaria transmission is challenging due to the complex and often nonlinear relationships between land use and malaria^38^.

Some studies have attempted to integrate land use and land cover into SIR models of malaria transmission. For example, a study in the Brazilian Amazon used simulations of land use changes that incorporated economic and epidemiological variables into the SIR framework^39^, this study highlights the potential value of integrating land use and land cover variables into SIR models of malaria transmission. However, further research is needed to improve our understanding of the complex relationships between land use, climate, and malaria transmission, and to develop more accurate and comprehensive models that can inform effective malaria control strategies^3,40,41^.

In addition to the use of mechanistic models like SIR Ross Macdonald (SIR model specific for malaria) models for evaluating the impact of malaria control and prevention measures, it’s also crucial to consider the significance of various seasons in the yearly cycle of *Anopheles*, which can elevate the risk of humans being bitten by *Plasmodium*-carrying mosquitoes^42^. While changes in climatic variables such as rainfall and temperature can create new breeding sites for mosquitoes and affect their behavior and life cycle, it may take some time for these changes to be reflected in changes in malaria incidence^43^, for example, changes in rainfall can create new breeding sites for mosquitoes^44^, but it may take several weeks for these sites to become fully functional and for mosquito populations to increase^45,46^. Therefore, understanding the temporal dynamics of climate-malaria relationships is essential for developing effective malaria control strategies that take into account the lag time between changes in climate variables and changes in malaria incidence in the local context^47–49^.

As a result of the necessity to examine the context of the elevated risk of malaria transmission in the Colombian Amazon, stemming from the rapid pace of land use change, the malaria habitat suitability and the need to carry out predictive models based on climatic seasons at fine scales, our main aim was to assess how the transmission of malaria is affected by precipitation, temperature, and changes in land use in a municipality in the Colombian Amazon. The study intends to determine the impact of these factors on malaria transmission while also considering the time lag effect. Additionally, the research will analyze two hypothetical scenarios (one pessimistic and the other optimistic) to evaluate the influence of managing and controlling some entomological–epidemiological variables on the occurrence of malaria cases in humans. By exploring this relationship, this investigation highlights the importance of considering multiple variables when studying complex systems, such as malaria transmission dynamics, and how the findings can contribute to developing evidence-based policies to improve public health outcomes.

## Materials and methods

### Study area

In relation to the occurrence of malaria instances within the Colombian Amazon, the chosen research site was the municipality of La Pedrera in the Amazonas Department. This choice was made for various reasons, including the notable increase in malaria cases in the area over the past decade^5^.

**Figure 1:**
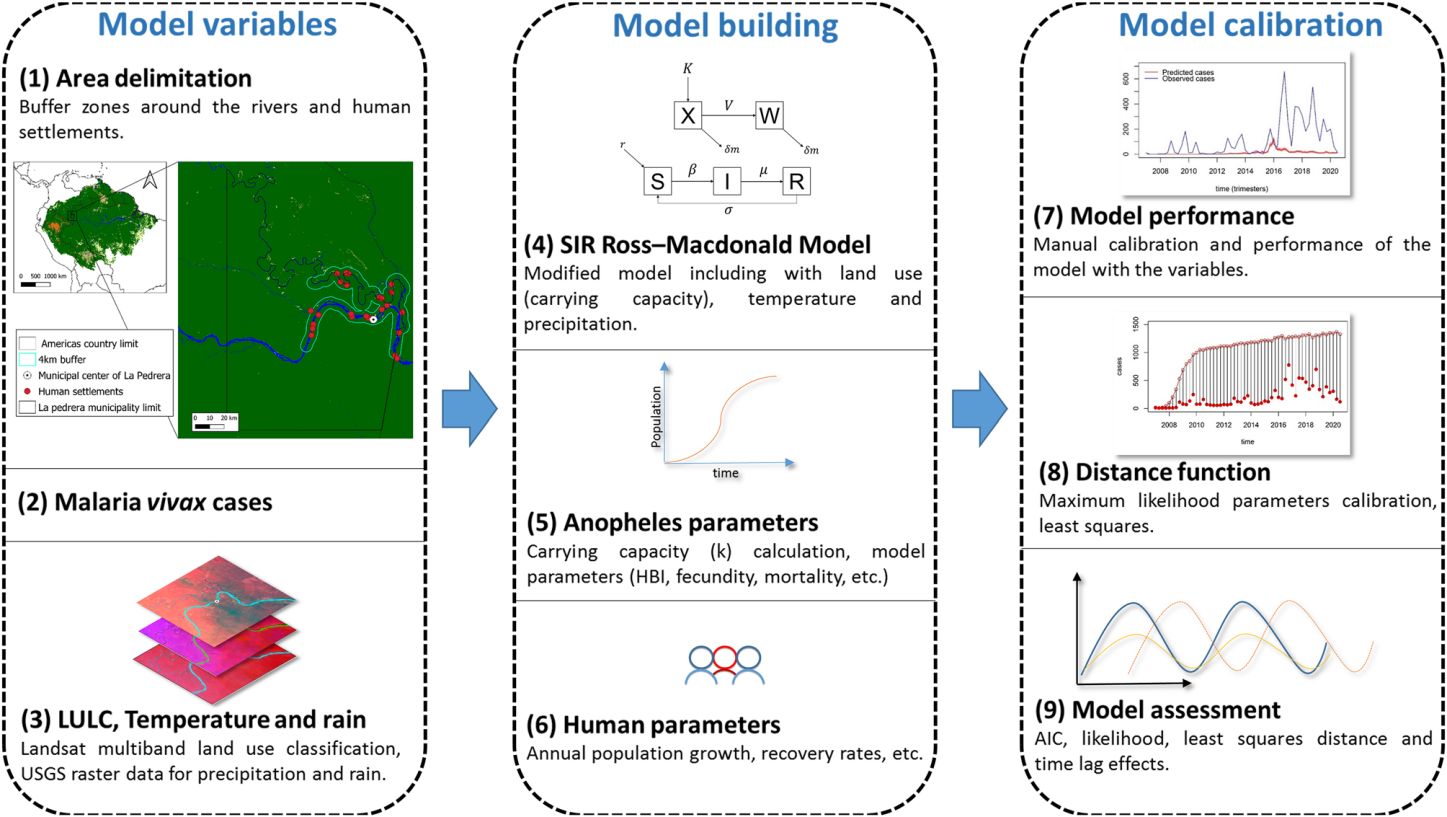
Flow chart indicating each of the steps used in the analysis, categorized into three main parts: model variables, model building, and model calibration. The landscape component is represented by steps 1, 3, and 5, while steps 2, 4, 5, 6, 7, 9, and 10 pertain to the eco-epidemiological aspects. (1) Location map of La Pedrera municipality: The Amazon biome from Mapbiomas in 2018^51^, along with the countries in which it is located. The inset map shows a 4 km buffer around the rivers where populations are concentrated, marked in aquamarine and red dots. The white dot with a black center denotes the location of La Pedrera’s municipal center, which is home to the hospital where most malaria cases are reported.

La Pedrera municipality is located in the department of Amazonas in the southern Colombian region with an area of 13,945 km^2^ (see Fig. 1), it borders Brazil in the east and almost all of its territory is made up of tropical forest, in addition to evergreen forest, as is common in the Amazon region, there are also flooded forests by the seasonal flooding pulse of the Caquetá river and the Apaporis river^50^. Indigenous communities and non-indigenous settlers are mainly located on the river’s edges due to the ease of access (see Fig. 1).

The following ethnic groups are present in the region: Yucunas, Macunas, Matapí, Tanimucas, Mirañas, Letuamos, Carihonas and Cubeos^52^. The main economic activities developed in the region are artisanal fishing, hunting, cassava, banana, corn and fruit crops, cattle and chickens to a lesser extent^53^, and in some surrounding places including Brazil, illegal mining^45^.

Within the realm of the Amazon biome, the pace of deforestation in Brazil's forest is strikingly rapid^54^. This trend is not exclusive to Brazil, as Colombia is also grappling with significant deforestation pressures, notably concentrated in the headwaters of macro-basins^55^. This occurrence is contributing to a reduction in the quality and quantity of water bodies^56^. The central challenges to the Amazon forest arise from human activities, with illicit coca cultivation and agricultural practices emerging as the primary catalysts for deforestation within the transition zone that spans the Andean, Orinoco, and Amazon biomes^57^. It is worth highlighting that the central region of Colombia, along with the northern zone of the Amazon biome, has borne the brunt of substantial loss in primary forests over the past two decades^58^. These activities are linked to increased deforestation rates and have significant ecological and social consequences.

This interconnected web of deforestation, environmental change, and social dynamics underscores the complex landscape of challenges facing the La Pedrera municipality, where addressing malaria cases and broader conservation efforts require a multifaceted approach^58^. Moreover, the presence of indigenous communities lacking access to proper healthcare facilities in the region further elevates the vulnerability of this municipality to malaria^59^. Consequently, the municipality requires the implementation of prevention and control strategies as it ranks high on the priority list for such plans^60^. Additionally, challenges related to accessing healthcare services and implementing preventive measures are notable in this context^61^.

### Malaria cases and population data

Human diary malaria cases of La Pedrera municipality from 2007 to 2020 were provided by Colombia's National Public Health Surveillance System^62^, due to the specificity of the parameters used in the modeling and the large proportion of *vivax* malaria cases in the Colombian Amazon, malaria cases were taken into account only from *Plasmodium vivax* infections. However, it’s essential to acknowledge certain limitations associated with this data source. First, there may be an underreporting rate of malaria cases, as not all individuals with malaria symptoms may seek medical attention or be included in the surveillance system’s records. Secondly, errors in the identification of the parasite in blood smears can occur, potentially leading to misclassifications or missed cases. Thirdly, the dataset includes reported cases without differentiation between new infections and cases with a possible origin of recrudescence.

The cases were downloaded without stratification by age, sex, or race. The total annual population size was downloaded directly from the National Administrative Department of Statistics^63^, which provides a population annual growing rate.

### Land use land cover, temperature and precipitation

Raster maps of land use were created from multiband Landsat image collections 5, 7, and 8 with 30 m cell-size. A supervised classification of remote sensing images was then performed using the random forest algorithm with 500 training samples and 10 trees. The choice of these values was carefully considered, striking a balance between precision and processing time/capacity. It’s important to note that as the number of training samples and trees increases, the accuracy of the maps improves, but this also leads to longer processing times for each set of bands. Therefore, the selected values represent an intermediate point that optimizes precision while ensuring attainable processing time.

To achieve this classification, we utilized a training layer containing 30 polygons for each land use type detected, including forest, flooded forest, sand, urban infrastructure, secondary vegetation, and water. These polygons enhanced the contrast and refined the spectrum intervals of land uses by generating various band combinations. However, the classification did face challenges in areas with uncertainty, typically occurring in intermediate pixels or at intersections between different land uses. These uncertain areas, whether due to land use transitions or tenuous cloud cover, were excluded from the total quantification to ensure the accuracy of the final results.

This approach allowed for the classification of different land uses in the study area. Nonetheless, due to the persistent high levels of cloud cover throughout the year, we generated yearly mosaics by combining multiple classified raster images to maximize the input data quality and produce a consensus map of yearly land use spanning from 2007 to 2020.

The satellite layers were acquired using the La Pedrera municipal center as the central reference point. A buffer of 50 km was established around this point, which is the farthest distance between the municipal center and the last registered human settlement in this area. To detect changes in land use in areas where human activity has taken place, an additional buffer of 4 km was applied within the 50 km buffer, specifically along the edges of the Caquetá and Apaporis rivers, where the present communities are located. This chosen overlapping region of the buffers served as our specific study area for collecting climate data and documenting alterations in land use (see Fig. 1).

Furthermore, acknowledging the significance of the water body edges for mosquito breeding, we calculated the water edge area (water cover edge length multiplied by 30 cm) to identify potential temporal and permanent mosquito spawning sites^46,64^. Finally, monthly air temperature (°C) and precipitation (mm) data were obtained from the Copernicus Open Access Hub platform between 2007 and 2020, utilizing data sourced from the Sentinel-2 satellite for the study area.

Subsequently, the temperature was averaged for each quarter of the year and the precipitation added due to the time scale used in the model for the following reasons: Firstly, a quarterly model strikes a balance between accuracy and practicality^65^ while daily or even monthly models may offer higher resolution and more precise results, using daily models may lead to issues with optimizing parameters reliably, producing less dependable long-term trends and patterns, and requiring overly complex and computationally intensive parameter optimization processes^66^. On the other hand, semi-annual models may not capture the fluctuations in disease dynamics as accurately as quarterly models, quarterly models can capture seasonal variations in disease transmission such as temperature and precipitation pikes^67^. This can be particularly useful for monitoring the effectiveness of interventions or identifying outbreaks before they become severe^68,69^.

### SIR Ross–Macdonald model

We propose a modification to the Baeza et al., model, our model focuses specifically on the transmission of *Plasmodium vivax* by *Anopheles darlingi* mosquitoes. The modified model consists of five compartments, including two for the vector mosquito: healthy $X$ and infected $W$ where $Nm=Totalmosquitoes(X+W)$**,** and three for humans: susceptible $S$, infected $I$, and recovered $R$ where $Nh=Totalpeople(S+I+R)$.

$$\frac{\delta S}{\delta t}=r\times Nh+\sigma \times R-\beta \times S,$$

$$\frac{\delta I}{\delta t}=\beta \times S-\mu \times I,$$

$$\frac{\delta R}{\delta t}=\mu \times I-\sigma \times R,$$

$$\frac{\delta X}{\delta t}=Fe\times Nm\left(1-\left(\frac{Nm}{Ktotal\times prec\times 0.01}\right)\right)-V\times X-\delta m\times X,$$

$$\frac{\delta W}{\delta t}=V\times X-\delta m\times W.$$

In this model, susceptible people become infected at rate

$$\beta =\left({a}_{u}\times prec \times 0.01\right)\times b\times \left(\frac{W}{\left(Nm\right)}\right)\times \left(\frac{Nm}{Nh}\right).$$

And healthy mosquitoes become infected at rate

$$V=\left({a}_{u}\times prec \times 0.01\right)\times c\times \left(\frac{I}{\left(Nh\right)}\right).$$

where a_u_ is the biting rate described by the Human biting index $HBI$

$$a_{u} = Biting{ }rate = \frac{HBI}{\frac{1}{BMDR}}.$$

And the BMDR is the Blood Meal digestion rate as a function of the temperature (T) described by the equation of the curve estimated from the data of Refs.^70–72^ on the gonotrophic cycle of *Anopheles albimanus* due to the absence of these data for *Anopheles darlingi*

$$BMDR\left( T \right) = \exp \left( {0.014 \times T} \right) - \exp \left( {0.014 \times 37.98 - \frac{37.98 - T}{{0.83}}} \right) - 1.144.$$

The parameter $r$ is the human birth rate or population growth rate. Infected humans recover at the rate $\mu$ and lose the immunity, then reentering the susceptible population at the rate $\sigma$. The parameter $b$ represents the probability of human being infected from infectious mosquito bite, and $c$ represents the transmission efficiency from infected humans to uninfected mosquitoes. The mosquitoes fecundity is described by the rate $Fe$ and it’s mortality by the rate $\delta m$.

In order to incorporate the effect of the landscape modification through the land use land cover change in the model through the time, we assigned a value of total mosquitoes per area unit (square meters) where each land use land cover type ($$landcovern$$) can carry a maximum mosquitoes number ($$carrying capacity value$$), thus, the total carrying capacity $${K}_{TOTAL}$$ was calculated yearly based on the total sum of hectares of each land cover multiplied by its carrying capacity value reported in the Table 1.

**Table 1:** The carrying capacity values calculated for each of the land uses in La Pedrera, Amazonas.

LULC type	Carrying capacity value (mosquitoes/m^2^)	% based on forest formation	References
Forest formation	0.01	100	^49,73^
Flooded vegetation	0.015	150	^74–76^
Open areas (secondary vegetation)	0.03	300	^12,19,23,77^
Urban infrastructure	0.015	150	^12,19,23,77^
Water edge	0.02	200	^46,78,79^

Land uses such as sand, bare soil, or water cover were excluded from the calculation since they do not play a significant role in the biological cycle of mosquitoes. Areas with strong currents or the central zones of lagoons were omitted from the analysis due to their minimal impact on the biological life cycle of mosquitoes due to the fact that the predominant breeding sites exist primarily at the perimeters of rivers, lakes, and other water bodies (These peripheral areas were quantified as “water edge”). The proportional percentage calculation is based on the forest formation because it is the natural habitat where *Anopheles darlingi* typically develops and the carrying capacity values are reported. Mosquitoes’ values are based on sampling methods and environmental covariates from satellite sources to estimate adult mosquito abundance per unit area^39^. Percentages greater than 100 indicate areas where mosquito populations can reproduce more easily due to greater food availability and fewer predators, resulting in a higher carrying capacity.

*m*^*2*^ square meters, *%* percentage based on the maximum carrying capacity of *Anopheles darling.*

$${K}_{TOTAL}=\sum_{i=0}^{n}\left(landcovern\times carryingcapacityvaluen\right).$$

Consequently, the model accounts for temperature and precipitation by factoring in their impact on the biting rate a_u_. Moreover, the model considers the effect of precipitation on the ecosystem's carrying capacity, which influences the development capacity and mosquito life cycle during rainy conditions. This assumes that as precipitation increases, the carrying capacity for each land use land cover type also increases.

### Vector parameters and human parameters

In order to match the simulated values with the observed cases, we estimated both the epidemiological and human parameters. Changes in these parameters affect the behavior of the simulated curve, so ranges were defined for each parameter. The optimization algorithm adjusts the parameters listed in Table 2, along with their bibliographical references. We assumed unreported values fall within logical and positive intervals based on appropriate epidemiological criteria.

**Table 2:** Epidemiological parameters for human host and mosquito vector.

Parameter estimated	Range	Unit	References
Sigma $\sigma$	[0–30]	Number of people/quarter	^80–82^
b	[0.01–0.6]	–	^17,83^
HBI	[0.1–1.0]	[% blood from humans]	^84,85^
Fe	[50–150]	[larvae/female]	^86^
c	[0.01–0.55]	–	^17,87^
$\delta m$	[12–30]	[days]	^88–90^
Mu $\mu$	[0–100]	Number of people/quarter	^17^

Parameters related with the ecology and vectorial capacity of *Anopheles* mosquitoes. The majority of the values for these parameters are obtained through empirical measurements and statistical inference methods, while the parameters related to human epidemiology processes are based on the literature and incorporate measurements from controlled experiments, as well as estimates from transmission models that are fitted to time series data on malaria cases in India and Africa.

Sigma ($\sigma$): Immunity human loss rate, b: Probability of human being infected from infectious mosquito bite, HBI: Human biting index, Fe: *Anopheles* females’ fecundity (eggs laid and retained), c: Transmission efficiency from infected humans to uninfected mosquitoes, Mu ($\mu$): Human recovery rate and δm: Mosquito mortality rate (day).

### Model assessment

The model was run by considering the accumulated cases of malaria caused by *P. vivax* per quarter, mean temperature, sum of precipitation, and annual carrying capacity value. After running the model, manual calibration of the parameters was done to verify changes in the behavior of curves of both state variables of mosquitoes (**X**, **W**) and humans (**S**, **I**, **R**). The calibration was done using the maximum likelihood distance function with a Poisson distribution of errors and a standard logarithm function link to compare the simulated and real cases of infected people (**I**) in the municipality of La Pedrera. The BFGS (Broyden–Fletcher–Goldfarb–Shanno algorithm) or quasi-Newton method was used to estimate the optimized parameters within the previous defined range.

To evaluate the influence of the temporary lag, four models were simulated based on parameter estimation using the maximum likelihood distance function. These simulations include: (1) No temporary lag, (2) One month of temporary lag, (3) Two months of temporary lag, and (4) Three months of temporary lag, using the values of the covariates 1, 2 and 3 months before and thus recreate the delayed environmental effect on the transmission of cases in the region. The best model was selected based on the log likelihood value, AIC, and visual assessment of fit between the observed and simulated cases.

We created two hypothetical scenarios to examine the effect of certain parameters on malaria cases and the potential impact of a positive intervention on transmission in the region. These models were based on the model parameters that most closely aligned with the observed data. The first scenario (negative scenario or pessimistic) involved increasing certain parameters that are positively associated with transmission by 5% (b, HBI, Fe, c) and decreasing parameters that have a negative relationship with transmission by 5% ($\delta m$). The second scenario was the opposite (positive or optimistic scenario), involving a decrease in parameters that are positively related to transmission and an increase in those that are negatively related to transmission.

Finally, the elaboration of the land use maps was carried out with QGIS 3.10.14-A Coruña, and the Semi-automatic Classification Plugin (SCP) 7.10.6—Matera^91^ coupled with ESA SNAP Desktop implementation 9.0.0 software. The differential SIR model equations were solved using deSolve 1.33 R package^92^, the maximum likelihood distance function using the bbmle 1.0.25 R package^93^, the parameters optimization and the graphs with R 4.2.1 in-built environment packages.

### Informed consent

In this study, studies were not carried out directly in humans, however, the data for the construction and evaluation of the model was based on observed cases for the municipality of La Pedrera—Amazonas Colombia. Available for free access on the SIVIGILA–COLOMBIA platform (https://portalsivigila.ins.gov.co/).

## Results

In the course of the analysis spanning from 2007 to 2020, a total of 6076 *P. vivax* cases were recorded within the municipality. Malaria cases exhibited a cyclical pattern, with the highest incidence ranging from 150 to 200 cases before 2015. The most notable peak in cases occurred during the third quarter of 2016, reaching 658 cases, followed by a decline in 2017, recording 382 cases during the second quarter. Another peak emerged in the third quarter of 2018, with 537 cases, after which the numbers dwindled to 13 in the second quarter of 2020.

**Figure 2:**
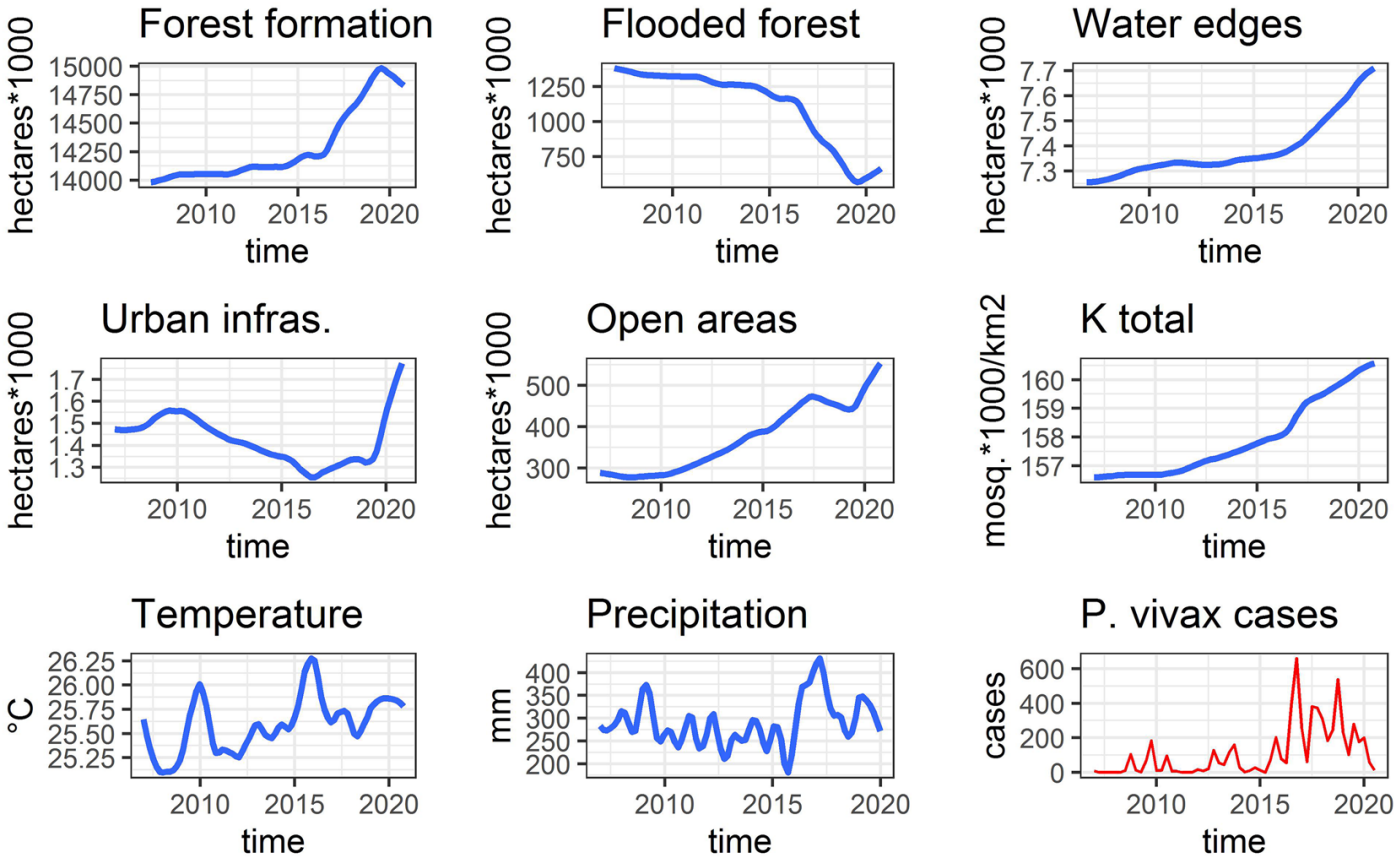
Variables of annual land use, carrying capacity, mean quarter temperature, quarter total precipitation and cases of *vivax* malaria in La Pedrera Amazonas in red. The y axes of the land use and carrying capacity (K) variables were transformed to facilitate their visualization. The land use variables were based on the supervised classification of remote sensing images (Landsat 5, 7 and 8), the temperature and precipitation were based on the Copernicus Open Access Hub platform between 2007 and 2020 and finally the carrying total capacity based on the total sum of hectares of each land cover multiplied by its carrying capacity value reported in the Table 1.

Regarding landscape modifications, there was no discernible trend or concentration of continuous deforestation, except for the flooded forest, which experienced a consistent decline throughout the observed area over the years. Between 2007 and 2020, significant changes in land use unfolded. Forested areas expanded during this period, with a minor reduction at the outset of 2020. In contrast, the flooded forest exhibited a continuous decline, intensifying since 2017. Areas surrounding bodies of water displayed a steady increase during the analyzed years. Urban infrastructure underwent cycles, with an upward trajectory until 2010, followed by a decrease until 2016, and a subsequent significant surge until 2020. Open areas exhibited constant expansion until 2017, followed by a temporary reduction, only to resume a positive trend in late 2019 (see Fig. 2).

Despite the absence of a consistent upward trend in urban infrastructure (in fact, more than half of the analyzed time frame witnessed a decrease), there was a constant increase in open areas, indicating the transformation of forests into grasslands and predominantly open herbaceous vegetation. The increase in forest, as quantified in the land use maps, was not a true increment but rather a shift from flooded forest to non-flooded tropical forests that were not necessarily located in high areas outside the area of the pulse of river flooding (a transition from Varzea forest to mainland forest). In general, all of these landscape modifications collectively resulted in a continuous rise in the presumed limit carrying capacity of *Anopheles darlingi*, with a more pronounced growth in 2016.

Moreover, temperature and precipitation variables exhibited a fluctuating pattern that corresponded to rainy and dry seasons, respectively. The precipitation had its highest peak in 2017-I, beginning to increase significantly since the start of 2016. On the other hand, the temperature reached its peak in 2015-IV, apparently with the same cyclical pattern of malaria cases but with a difference in time in onset or delay effect (the climatic events of peaks in temperature and precipitation occurring first than the peaks of malaria cases). See Fig. 2.

### Model performance and time lag effect

**Figure 3:**
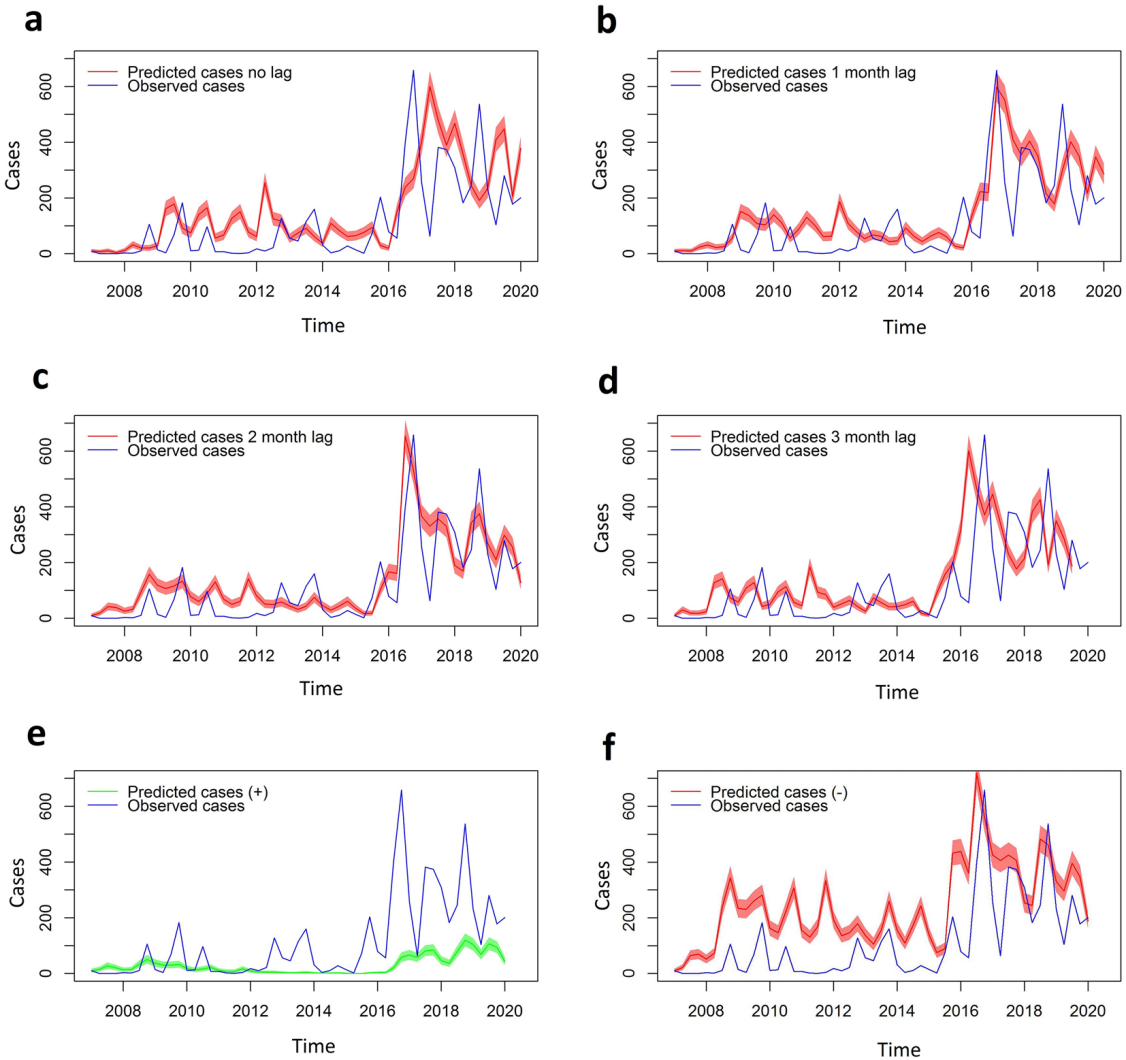
Infected people/per semester (simulations in red and green line with confidence interval) and infected people (observed cases in blue line) for the six models. (**A**) No temporary lag, (**B**) 1-month temporal lag, (**C**) 2-month temporal lag and (**D**) 3-month temporal lag, (**E**) optimistic scenario and (**F**) pessimistic scenario. The pessimistic and optimistic scenarios were based on the parameter modifications from the 2-month temporal lag model (the best fit model).

The results of our study showed that the 2-month lag model had the best fit based on both the Akaike Information Criterion (AIC) and the maximum log likelihood (see Table 3). Taking into account the visual assessment of the adequacy of the model, the 2-month lag model was able to more accurately track the peaks and valleys observed in the actual cases, specifically the highest peak of 2016-III quarter, the second peak of 2018-III, quarter and the downward trends following seasonal peaks of infection. In contrast, the other models, which lacked a temporal lag or had 1 or 3 months of temporary lag, did not capture the observed pattern of infections. The second-best fitting model was the 1-month temporal lag model, which displayed good agreement with the maximum peak of cases 2016-III and 2018-III but showed asynchrony in all other peaks of cases (see Fig. 3). While the 2-month temporary lag model exhibited a superior fit during certain time periods, it failed to accurately track the observed cases between 2010 and 2012 and also underestimated the magnitude of the peaks between 2012 and 2014, which were higher in the observed cases than in the simulated cases (see Fig. 3).

**Table 3:** AIC criteria information, maximum log likelihood of the models to evaluate the effect of the temporary lag with the optimization of parameters based on the observed cases.

Model	Max log likelihood	AIC
No temporal lag	6325.302	6339.302
1-month lag	4847.832	4861.832
**2-month lag**	**2805.243**	**2819.243**
3-month lag	5317.259	5331.259

Best model fit in boldface.

Regarding the simulated models that were created by modifying the adjusted parameters, the negative scenario, which involved a 5% reduction in the parameters, resulted in malaria cases increases of up to approximately 300% in some periods (i.e., 2009 and 2010), with the maximum peak numbers similar to those observed in 2017 and 2019, without the 300% increase seen in the earlier periods. Despite these changes, the negative scenario still exhibited a cyclical behavior with significant seasonal peaks and valleys (see Fig. 3, graph E and F).

Conversely, the modifications made for the optimistic scenario demonstrated that a 5% reduction in parameters could result in a complete elimination of cases between 2013 and 2016, despite a 31% probability of infection from infected mosquitoes, 22% of HBI and only a 5% reduction in mosquito mortality (see Table 4). For the most significant peaks of observed cases, the optimistic scenario resulted in a reduction from approximately 650 cases to around 100 cases, leading to a maximum peak of approximately 160 cases in 2019. Ultimately, the simulated cases for the optimistic scenario deviated from their previous cyclical nature with marked peaks and valleys.

**Table 4:** Two month’s lag model parameters calculated from the maximum likelihood (the best fit model) and simulated positive and negative scenarios.

Model	Parameters						
	Sigma $\sigma$	b	HBI	Fe	c	Mu $\mu$	$\delta m$
2-month lag	5.0000004	0.3268949	0.2355878	116.84353	0.2608142	2.9999991	14.999997
Optimist scenario (+)	4.7500003	0.3105501	0.2238084	111.00135	0.2477734	3.1499990	14.249997
Pessimistic scenario (−)	5.2500004	0.3432396	0.2473671	122.68570	0.2738549	2.8499991	15.749996

The parameters used to simulate both positive and negative scenarios were modified by increasing or decreasing them by 5% depending on their influence on malaria transmission.

Sigma ($\sigma$): Immunity human loss rate, b: Probability of human being infected from infectious mosquito bite, HBI: Human biting index, Fe: Anopheles females’ fecundity (eggs laid and retained), c: Transmission efficiency from infected humans to uninfected mosquitoes, Mu ($\mu$): Human recovery rate and δm: Mosquito mortality rate (day).

## Discussion

The study provides valuable insights into the relationship between climatic and land use factors and malaria transmission in La Pedrera. The results suggest that the model was able to effectively replicate the observed malaria cases by taking into account the cyclical patterns in temperature-precipitation and the increasing trend in mosquito carrying capacity due to changes in land use. The study also highlights the importance of considering the duration of ecological and biological processes, such as the mosquito’s biological cycle after peak rain or temperature, and the parasite’s development time within mosquitoes and humans (2 months), thus the findings suggest that elevated levels of precipitation and temperature increase the risk of malaria infection in the subsequent two months. Overall, this study highlights the potential of climate and land use models in predicting and preventing malaria outbreaks in similar contexts.

In relation to climatic factors, the El Niño Southern Oscillation (ENSO) climatic phenomenon plays an important role in the transmission of malaria in the Colombian Amazon region^31,94^. The patterns of high temperature (year 2015) and subsequent increase in rainfall (year 2016), which are directly related to the peak of infections observed in the year directly related with the simulations, are determined by the ENSO phenomenon^95^. The study also reveals the impact of climate change on the rising cases of malaria in the Amazon region, as there has been a temperature increase of 0.5 °C from 1983 to 2016^96^, leading to abnormal weather patterns that represent a greater risk for malaria transmission. Although the time window analyzed in the study is not sufficient to confirm the temperature increase, it does show a cyclical pattern that is increasing according to reported data on temperature increases in the Amazon possibly due to climate change^97^.

The relationship between land use and precipitation can be explained biologically, as different land uses with their carrying capacities are enhanced by precipitation^98^. This is particularly important for water bodies, temporary and permanent wells in the forest, urban infrastructure, and secondary vegetation, which are all important breeding sites for mosquitoes^99^. However, it is also important to note that rain can have a limiting effect on mosquito populations when it exceeds a certain threshold, as it can wash away breeding sites or be deadly to adults who lack a suitable place to hide^100,101^.

It is important to note that land use changes have an impact on *Anopheles* populations’ carrying capacity, not only in forest or flooded the rising forest areas, but also in other landscape characteristics such as the rising of water body edges and open areas with deforested grazing or shrubby vegetation where people reside (see Fig. 2). In Belize, a comparable pattern was identified in which transitioning from native vegetation to a mixture of crops and marshes is linked to elevated populations of *Anopheles* spp.^102^. This highlights the multi-biological nature of malaria, where not only the presence of high-risk land uses is significant, but also the way humans access and modify them, which can either increase or decrease the risk^103^.

The model was able to track the observed cases’ behavior. However, there were time periods where cases were underestimated (2013–2014). This may be due to variables not considered by the model, such as migration, mining, transmission due to other important vectors^7^ and recrudescence. Firstly, La Pedrera is located on the border of Brazil, and there is frequent migration between the two countries, especially because the area is predominantly indigenous, and political-administrative boundaries do not necessarily apply to their distribution area^16,104^. Additionally, gold mining is a prevalent undertaking in the neighboring areas, exposing individuals to an elevated risk of malaria transmission. Prolonged exposure to water bodies and residing in camps lacking protection measures against mosquitoes or access to healthcare services contribute to this heightened vulnerability^45,105^. Illegal miners may introduce malaria cases to La Pedrera from other regions via the Caquetá and Apaporis rivers, which are not necessarily related to the activities in La Pedrera. Lastly, *Plasmodium vivax* and *Plasmodium ovale* can remain latent in the human body, and incomplete antimalarial drug treatment may not entirely eliminate the parasite from the body^106^. As a result, people may experience symptoms that are not necessarily caused by new infections^107,108^.

On the other side, during the periods when the model indicated a higher number of cases than those observed (2010–2012), we believe that several factors may have contributed to this discrepancy. Firstly, the underreporting of data could be a possible reason, given that the region’s health services are still underdeveloped and largely managed by health campaigns from the capital city of Leticia—Amazonas without adequate oversight^109^. Secondly, acquired immunity to malaria could also be a contributing factor. It has been previously reported that malaria cases in endemic areas are influenced by resistance built up through constant infections over the years^106^. Lastly, there are various complex limiting environmental factors that affect the vector’s development, which are challenging to model, these include non-constant mortality rates, ecological interactions with other species that alter their population size, human population not homogeneously mixed and other variables that could improve the mathematical framework and the real cases simulations^36^.

Regarding the impact of the parameters derived from optimizing the 2-month temporary lag model, we can identify several factors related to environmental variables or intrinsic immunological factors and parasite load. For instance, the human biting index (HBI) is associated with temperature due to the mosquito's metabolic cycle, while precipitation and land use affect the probability of mosquitoes feeding on humans. Mosquito fecundity is influenced by temperature, availability of food, and suitable bodies of water for oviposition. Mosquito mortality, on the other hand, is primarily affected by age, environmental stressors, and exposure to insecticides^88–90^.

Intrinsic human parasitic or immunological factors of mosquitoes determine the parameters such as b and c, that are more closely related to the pathogen load in the human host’s blood, the immune response of the mosquito, the duration of the blood meal, the mosquito’s age and infection status, and the immune response of the human host^17^. Therefore, this model, despite having calculated both epidemiological and entomological parameters such as human recovery and re-entry rates, highlights the significance of HBI, fecundity, and mosquito mortality rates, which are the parameters most affected by changes in the environment, such as increasing temperatures, precipitation, and land use changes, which in turn increase the likelihood of infection.

Nonetheless, this does not imply that intrinsic-immunological and epidemiological parameters are insignificant or unimportant for control plans, which is where positive-optimistic and negative-pessimistic scenarios come into play. Parameters such as b, c, Mu (μ), and even δm can be manipulated to significantly reduce the number of cases. Traditional control measures such as wearing appropriate clothing in high-risk biting areas, using mosquito nets, improving the implementation and efficacy of antimalarial treatments, controlling mosquito breeding sites, and reducing mosquito fecundity could decrease both the efficiency of mosquito-human and human-mosquito transmission. The latter becomes critical due to the growing resistance of mosquitoes to insecticides. However, according to the findings of this study, it is crucial to consider that other variables can be modified with appropriate measures and time based on the temporary lag. Our findings support early prevention plans to take action and increase general awareness 2 months before expecting high numbers of malaria cases following high rainfall and temperatures.

The performance of the models highlights the significance of the temporal lag, and the 2-month model was found to be the most accurate. This finding is consistent with previous research, which demonstrated a relationship between precipitation and malaria cases, with cases being associated with precipitation occurring 9 weeks earlier (on average, 2 months) and capable of predicting both the magnitude and cyclical pattern of the disease^110^. Another study found that *Anopheles* populations were higher during the dry season^111^, which can be attributed to their development and oviposition during the preceding rainy season or transition from rainy to dry seasons. Therefore, it is important to consider different temporal lags for temperature and precipitation, as high rainfall promotes reproduction during the rainy season when temperatures are lower. In contrast, during the dry season, high temperatures directly impact the gonotrophic cycle, where the rate of digestion increases with temperature, resulting in a higher rate of human blood index (HBI) in adult mosquitoes already developed and not dependent on bodies of water for their initial cycle.

## Conclusions

The study highlights the significance of considering the cyclical patterns in temperature, precipitation and land use changes in predicting malaria transmission. The findings indicate that elevated levels of precipitation and temperature increase the risk of malaria infection in the following 2 months, particularly in areas with mixed secondary vegetation and urban infrastructure near primary forest formations or the edges of water bodies. Additionally, it is crucial to emphasize the model’s strong fit to the observed cases, which indicates that it may accurately forecast cases in areas with comparable socioeconomic and environmental conditions. The El Niño Southern Oscillation (ENSO) also plays an essential role in malaria transmission in the region. The analysis suggests that land use changes impact the carrying capacity of Anopheles populations, not only in forested areas but also in other landscape characteristics such as water body edges and urban infrastructure. Our findings highlight the multi-biological nature of malaria, where humans’ access and land use modification can modulate disease transmission risk. For future research, separating susceptible people into different compartments based on exposure and including other social variables, such as human migration, is recommended. These results could be valuable for public health officials and policymakers to develop effective strategies for early warning systems and malaria control in the region. Our results suggest continuously monitoring precipitation, temperature, and land use variables to predict high-risk areas can be beneficial to long-term disease mitigation.

Finally, and especially in the La Pedrera—Amazonas, it is important to consider the effects and direct impact of deforestation and modification of the Amazonian primary forest and the need for better malaria control measures. These measures include implementing plans to use mosquito nets in urban areas, controlling breeding areas in both open and urban areas, having prevention plans that involve wearing clothing to reduce mosquito bites in flooded forests or near bodies of water and improving the health system of the region by expanding the use of effective treatments against malaria, particularly after peak temperatures and rains. These control measures can help intervene in the eco-epidemiological parameters and have an impact on reducing malaria cases and improving the quality of life for the people, as observed in both pessimistic and optimistic simulated cases.

## Data Availability

Climatic data obtained from the Copernicus Open Access Hub platform (https://scihub.copernicus.eu/). Land use maps created from the Landsat collections 5, 7, and 8 layers for the studied area and downloaded directly using the free access Semi-automatic classification plugin for QGIS (https://plugins.qgis.org/plugins/SemiAutomaticClassificationPlugin/). Human malaria cases for each municipality available on the open access Colombian National Health Institute platform SIVIGILA (https://portalsivigila.ins.gov.co/).
